# Survival outcomes of patients with concomitant acute variceal bleeding and acute coronary syndrome, and the role of antiplatelet agents: an institutional experience from a lower middle-income Country

**DOI:** 10.1186/s12876-022-02611-4

**Published:** 2022-12-28

**Authors:** Shameel Shafqat, Ajeet Kumar Lohana, Rajesh Kumar Bansari, Om Parkash

**Affiliations:** 1 Medical College, The Aga Khan University, Karachi, Pakistan; 2 Section of Gastroenterology, Department of Medicine, The Aga Khan University, 74800 Karachi, Pakistan

**Keywords:** Acute coronary syndrome, Acute variceal bleeding, Anti-platelet agents, Cirrhosis

## Abstract

**Background:**

There is strong evidence demonstrating the incidence of Acute Coronary Syndrome (ACS) among patients with cirrhosis, with the initiation of antiplatelet therapy being subject to debate due to an increased risk of bleeding. This study aimed to determine mortality among patients presenting with concomitant Acute Variceal Bleeding (AVB) and ACS at Index admission. Furthermore, the recurrence of AVB and ACS among patients discharged with or without antiplatelet therapy was determined.

**Methods:**

This retrospective study was conducted at the Aga Khan University Hospital, Karachi, Pakistan on patients ≥ 18 years of age admitted to our ER with concomitant ACS and AVB between January 2002 to December 2017. Follow-up for 6 months or till death (if < 6 months), was observed, to help determine the incidence of recurrent AVB and ACS. The incidence of AVB and ACS was then compared amongst patient groups based on the usage of anti-platelet drugs on discharge.

**Results:**

A total of 29 patients were included, with a mean age of 58.7 ± 11.0 years. Seven patients died on admission, having worse underlying liver disease. No mortality was reported among the remaining 22 patients. All 22 patients underwent surveillance endoscopy with variceal band ligation until obliteration, as needed. Only 7 patients from the surviving cohort received antiplatelet therapy. After 6.05 ± 1.1 months of follow-up, 1/22 (4.5%) developed recurrent AVB and 2/22 (9.1%) developed cardiovascular events. Importantly, there was no significant difference in the incidence of recurrent AVB (*P* = 1.000) and ACS (*P* = 0.091), depending on the use of antiplatelet therapy.

**Conclusion:**

Concomitant AVB and ACS is a severe disorder with increased mortality among cirrhotic patients at presentation. The incidence of AVB does not seem to exacerbate with the use of antiplatelet agents, provided successful obliteration of varices is achieved using elective band ligation.

## Background

Liver Cirrhosis, previously theorized to have a protective effect from developing heart disease [1, 2], has been linked to having a strong association with Coronary Artery Disease (CAD) [3], with cirrhotic patients developing significantly increased CAD vs. patients with healthy livers (*P* = 0.001) [4]. Furthermore, liver transplant candidates have been found to have moderate to severe CAD (26.0-37.6%), depending on the diagnostic modality used [5–7].

Anti-platelet regimes have been the cornerstone to improve outcomes in patients with Acute Coronary Syndromes (ACS) secondary to CAD, albeit with an overall increased risk of bleeding [8, 9]. Hence, for cirrhotic patients already prone to bleeding, the decision to use anti-platelet therapy has been subject to debate in patients who present with concomitant Cirrhosis and ACS.

One of the most severe life-threatening complications of cirrhosis is acute variceal bleeding (AVB), which accounts for up to 70% of upper-gastrointestinal-bleeding (UGB) episodes amongst cirrhotic patients [10, 11]. The mortality rate after Index admission for AVB has been reported up to 50%, while mortality after rebleeding has been estimated at around 30% [12]. A study consisting of 148 patients analyzed the results of using Dual Anti-Platelet Therapy (DAPT) in patients who underwent Percutaneous Coronary Intervention (PCI) (Cases) and those treated with other medical therapies for CAD (Controls). DAPT was used in 98.5% cases vs. 5% controls. The incidence of UGB was significantly higher for cases vs. controls on follow-up at 2 years (27.9% vs. 5%; *P* = 0.0002). Up to 36.9% of UGB was attributed to esophageal varices and portal hypertensive gastropathy, suggesting an increase in rates of UGB secondary to varices in cirrhotic patients receiving DAPT [13]. In another study, Russo et al. reported that 12.5% of patients with Cirrhosis and CAD, on DAPT for 1 year, developed fatal variceal bleeding. This was in comparison to 6.3% of age and gender-matched controls who developed non-fatal variceal bleeding during follow-up on antiplatelet therapy (*P* = 0.86) [14].

Based on the above evidence, physicians are concerned regarding the initiation of antiplatelet therapy among patients who present with AVB and ACS simultaneously. Moreover, there is a paucity of published data regarding management of such patients within our region. Thus, we intended to determine the incidence of mortality at Index admission, and up to 6 months afterward, in cirrhotic patients presenting with concomitant AVB and ACS. Furthermore, we also determine the frequency of re-bleeding and/or recurrent coronary events in this high-risk patient cohort, discharged with or without antiplatelet therapy over a mean follow-up of 6 months.

## Methods

In this focused retrospective study, cirrhotic patients who presented with ACS and simultaneous AVB to the Aga Khan University Hospital (AKUH) between January 2002 to December 2017, were included. Exemption from the Institutions’ Ethical Review Committee was acquired, and data were collected on a pre-designed questionnaire on patients ≥ 18 years of age. The data was retrieved from the patient database using the ICD-10 coding system, exclusively extracting for cirrhotic patients with ACS and AVB.

Patients were included if AVB was confirmed on upper-gastro-intestinal endoscopy, and ACS, in the form of Acute Myocardial Infarction (AMI) [either ST-segment elevation Myocardial Infarction (STEMI) or non-STEMI (NSTEMI)], was present, defined as per the guidelines published in the European Heart Journal: AMI defined as an increase in high-sensitivity Cardiac Troponin T or I, with at least one value above the 99th percentile of the upper reference limit (0–57 ng/dl in males and 0–37 ng/dl in females) and at least one of the following: symptoms of myocardial ischemia, new ischemic ECG changes, development of pathological Q waves on ECG, etc.; NSTEMI patients may also exhibit ECG changes such as transient ST-segment elevation, persistent or transient ST-segment depression, T-wave inversion, flat T waves, pseudo-normalization of T waves, or ECG may be normal [15]. Patients with underlying hepatocellular carcinoma, acute on chronic liver failure (ACLF), end-stage renal disease, congestive cardiac failure, and any other malignant disorder which could affect the short-term (6-month) mortality, were excluded.

Records were reviewed until 6 months follow up, or till death (if < 6 months) from index admission, to determine the incidence of mortality at index admission, along with the incidence of recurrent AVB and ACS during follow-up. The incidence of recurrent AVB and/or ACS was then compared amongst patient groups based on the usage of anti-platelet drugs on discharge.

Categorical variables were presented as frequencies and percentages, and continuous variables as means with standard deviations, or medians with their respective ranges. Chi-squared test and Independent student t-test were used for further statistical analyses, which were performed using SPSS V25.0.

A *P*-value of < 0.05 was considered statistically significant throughout our study.

## Results

Twenty-nine patients were found to have presented with concomitant AVB and ACS over the study duration, predominantly male [19/29 (65.5%)] with a mean age of 58.7 ± 11.0 years. Twenty-five patients (86.0%), presented predominantly with UGB symptoms such as hematemesis and melena. Decompensated Cirrhosis was present in 19/29 (65.5%) patients and a total of 14/29 (48.3%) patients presented with Ascites, in which there was an equal distribution of 50% between mild-moderate and severe ascites. A majority of patients presented with NSTEMI [24/29 (82.8%)], followed by STEMI [5/29 (17.2%)]. Other baseline characteristics are presented in Tables 1 and 2. While patients were not administered Terlipressin secondary to the risk of Cardiovascular Ischemia [16], 24/29 (82.8%) patients received Octreotide (Somatostatin) as a complementary temporizing measure. Blood was also transfused to 27/29 (93.1%) patients, keeping a Hemoglobin threshold of < 10 for transfusion [17], with a median of 4.0 (1–14) units transfused; all patients then underwent a session of Esophageal Variceal Band Ligations (EVBL) at their initial admission.

**Table 1:** Characteristics of study population

Baseline characteristics	Total (*n* = 29)	Survivor group (*n* = 22)	Non-survivor group (*n* = 7)	Significance (*P*-value)
*Gender*				1.000
Male	19 (65.5)	14 (63.6)	5 (71.4)	
Female	10 (34.5)	8 (36.4)	2 (28.6)	
*Co-morbidities*				
Hypertension	22 (75.9)	17 (77.3)	5 (71.4)	1.000
Ischemic heart disease	13 (44.8)	10 (45.5)	3 (42.9)	1.000
Diabetes mellitus	19 (65.5)	16 (72.7)	3 (42.9)	0.193
Chronic kidney disease	1 (3.4)	0	1 (14.3)	0.241
*ACS*				0.569
STEMI	5 (17.2)	3 (13.6)	2 (28.6)	
NSTEMI	24 (82.8)	19 (86.4)	5 (71.4)	
*Etiology of cirrhosis*				
Hepatitis C	15 (51.7)	11 (50.0)	4 (57.1)	1.000
Hepatitis B	3 (10.3)	1 (4.5)	2 (28.6)	0.136
Alcoholic liver disease	2 (6.9)	1 (4.5)	1 (14.3)	0.431
NBNC chronic liver disease	9 (31.0)	9 (41.0)	0	0.066
*Ascites*				0.215
Present	14 (48.3)	9 (40.9)	5 (71.4)	
Absent	15 (51.7)	13 (59.1)	2 (28.6)	
*Size of varices*				0.052
Large	27 (93.1)	22 (100.0)	5 (71.4)	
Small	2 (6.9)	0	2 (28.6)	1.000
*Site*				
GOV/ IGV 1/ IGV 2	26 (89.7)	2 (9.1)	6 (85.7)	
Absent				0.410
*Active bleeding*	3 (10.3)	20 (90.9)	1 (14.3)	
Yes	16 (55.2)	11 (50.0)	5 (71.4)	
No	13 (44.8)	11 (50.0)	2 (28.6)	
*Decompensation*				0.367
Present	19 (65.5)	13 (59.1)	6 (85.7)	
Absent	10 (34.5)	9 (40.9)	1 (14.3)	
*Anti-platelet usage*				0.147
Yes	7(24.1)	7 (31.8)	0	
No	22 (75.9)	15 (68.2)	7 (100.0)	

All categorical variables are reported as numbers (percentages).

*UGB* Upper Gastro-intestinal bleeding,  *ACS* Acute coronary syndrome,  *GOV* Gastroesophageal varices, *IGV*  Isolated gastric varices, *AVB* Acute variceal bleeding. Chi-squared test was used for categorical variables. *P-values are significant (< 0.05)

**Table 2:** Baseline lab characteristics of study population

Baseline characteristics	Total (*n* = 29)	Survivor group (*n* = 22)	Non-survivor group (*n* = 7)	Significance (*p*-value)
Age	58.7 ± 11.0	58.6 ± 10.8	58.7 ± 12.5	0.987
*At presentation*				
Systolic blood pressure (mmHg)	122.3 ± 28.9	127.3 ± 31.3	110.3 ± 18.8	0.196
Diastolic blood pressure (mmHg)	72.5 ± 18.5	75.1 ± 20.0	66.3 ± 13.4	0.301
Pulse/heart rate (/min)	112.5 ± 17.5	110.5 ± 19.4	117.1 ± 11.8	0.413
Hemoglobin (Hb) (g/dl)	7.97 ± 2.0	7.75 ± 2.2	8.66 ± 1.5	0.313
INR	1.54 ± 0.8	1.31 ± 0.3	2.26 ± 1.3	0.095
Prothrombin time seconds)	16.8 ± 8.3	14.1 ± 3.6	24.9 ± 13.3	0.077
Total bilirubin (mg/dl)	3.17 ± 5.5	3.15 ± 6.2	3.21 ± 2.6	0.980
Albumin (g/dl)	2.72 ± 0.5	2.84 ± 0.5	2.37 ± 0.4	0.045*
Creatinine (mg/dl)	1.34 ± 0.8	1.27 ± 0.7	1.57 ± 1.0	0.378
Troponin (µg/dl)	18.1 ± 22.0	16.7 ± 18.9	22.5 ± 32.0	0.694
Child–PUGH score	8.76 ± 2.4	8.09 ± 2.1	10.8 ± 1.77	0.005*
Meld score	15.4 ± 8.8	13.4 ± 8.1	21.9 ± 8.0	0.023*
*Stay in hospital*				
Hb in ward (g/dl)	8.44 ± 2.3	8.22 ± 2.2	8.96 ± 2.7	0.493
Drop in Hb from baseline (g/dl)	1.53 ± 1.9	0.96 ± 1.6	2.91 ± 1.8	0.017*

All numerical values as Mean ± S.D. Independent student *t*-test for numerical variables. *P-values are significant (< 0.05)

A total of 7/29 (24.1%) patients died at Index admission. Two of the seven (28.6%) patients died secondary to cardiac causes: one due to heart failure, and the other because of bradycardia with underlying heart block. Two patients (28.6%) had fatal re-bleeding, one of whom was managed with Trans-jugular Intrahepatic Porto-systemic Shunt (TIPS), while the other patient was too critical to undergo any intervention. The remaining 3 patients (42.8%) died secondary to sepsis with multi-organ failure, comprising of respiratory (*n* = 2) and renal (*n* = 3) failure, and a brain infarct (*n* = 1). There was no mortality in the remaining 22 patients, within their 6 months of follow-up.

Baseline comparison between the surviving patient cohort (*n* = 22) and the patients with mortality (*n* = 7) revealed the underlying liver disease was worse in patients who died compared to the survivors, as shown with significantly higher CHILD-PUGH (10.8 ± 1.77 vs. 8.09 ± 2.1; *P* = 0.005) and MELD (21.9 ± 8.0 vs. 13.4 ± 8.1; *P* = 0.023) scores, as well as lower albumin (g/dl) at presentation (2.37 ± 0.4 vs. 2.84 ± 0.5; *P* = 0.045). Ascites was not found to play a significant role in determining mortality (71.4% vs. 40.9%; *P* = 0.215). Additionally, patients in the non-surviving cohort had a statistically significant higher drop in mean hemoglobin (g/dl) from baseline at Index admission than those patients who lived (2.91 ± 1.8 vs. 0.96 ± 1.6; *P* = 0.017). There was no other significant association between the two groups.

Among the patients who survived following Index admission, 7/22 (31.8%) received antiplatelet therapy in the form of a single antiplatelet agent (SAPT) (either Acetyl-Salicylic Acid (ASA) 75 mg or Clopidogrel 75 mg; Once Daily). The median time to start patients on their anti-platelet regime was 3.0 days (0–60.0), while the mean follow-up duration (months) was 6.05 ± 1.1 months. All 22 patients underwent surveillance endoscopy after 2 weeks of Index admission, and varices were band-ligated, with follow-up surveillance and further band-ligation until obliterated.

**Fig. 1:**
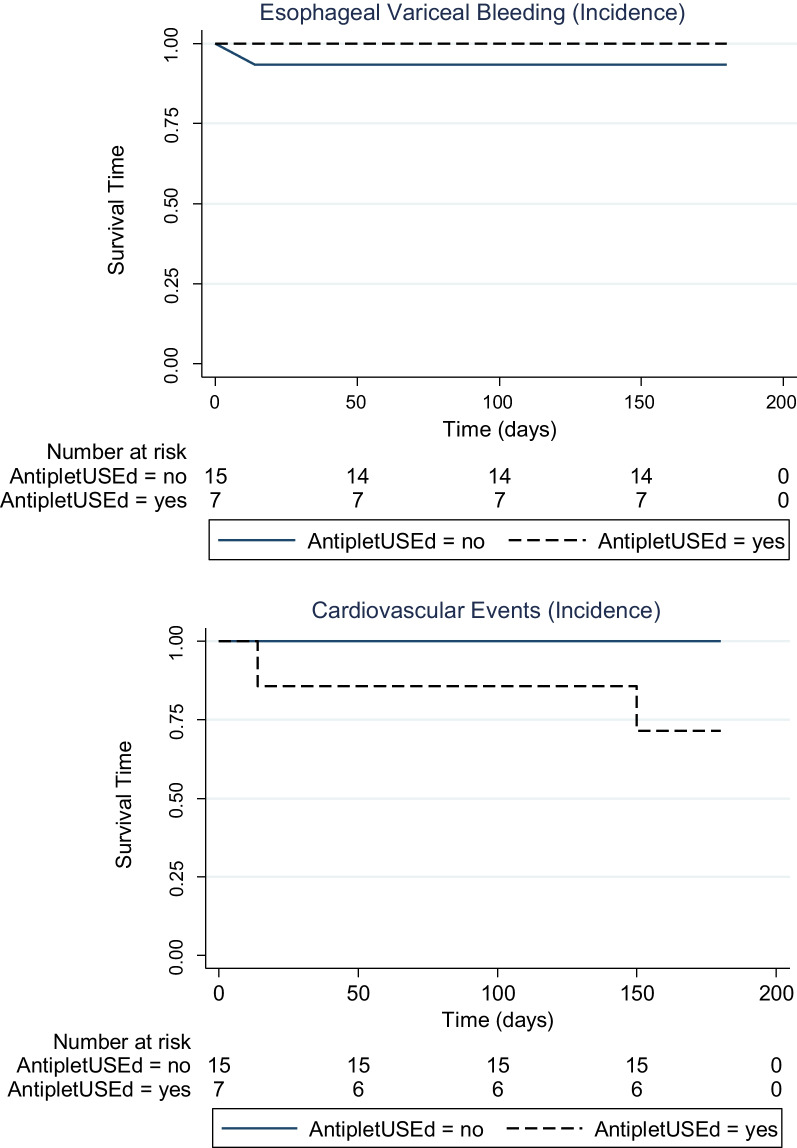
Incidence of Acute variceal bleeding (AVB) and Acute coronary events on follow up. One patient in the group without antiplatelet use developed recurrent AVB on day 10. Two patients with antiplatelet use developed acute coronary event; one at day 20, and other at day 150, on mean follow up of 180 days

**Fig. 2:**
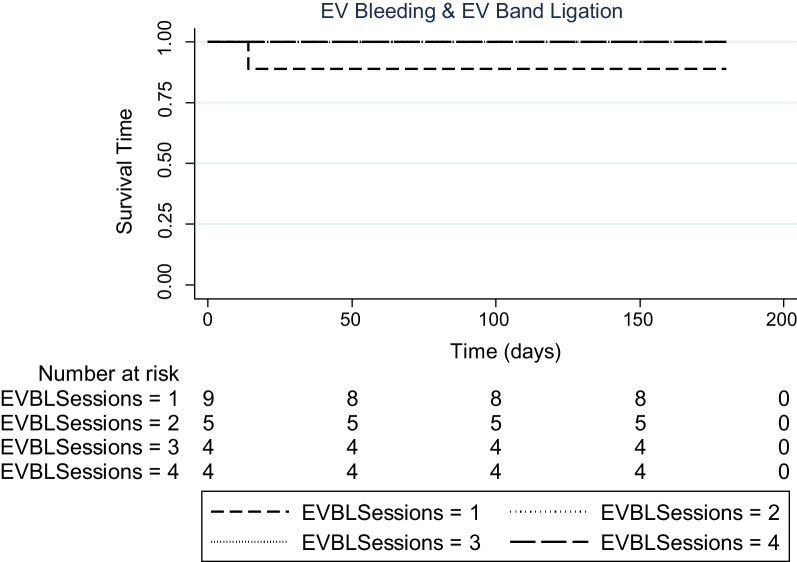
The recurrent Acute variceal bleeding (AVB) and frequency of Esophageal variceal band ligation (EVBL) sessions. There was only one recurrent AVB noted in the patient with one session of EVBL. No patient with ≥ 2 session of EVBL developed recurrent AVB

Only 1/22 (4.5%) patients developed re-bleeding from varices and 2/22 (9.1%) developed recurrent episodes of ACS **(**Fig. 1**).** Importantly, the incidence of recurrent AVB (*P* = 1.000) and ACS (P = 0.091) did not depend on the use of antiplatelet therapy **(**Table 3**)**. This episode of AVB on follow-up was noticed in a patient who only had a single session of EVBL. Of note, a total of 9 patients had only 1 session of EVBL, while the remaining 13 underwent ≥ 2 sessions of EVBL, including Index admission plus subsequent surveillance band ligation **(**Fig. 2**)**.

**Table 3:** Use of antiplatelet therapy in the Surviving Cohort

Follow-up	Usage of anti-platelets in survivor cohort (*N* = 22)		Significance (*p*-value)
	No (*N* = 15); *n* (%)	Yes (*N* = 7); *n* (%)	
*Episode of ACS*			0.091
Yes	0	2 (28.6)	
No	15 (100)	5 (71.4)	
*Episode of AVB*			1.000
Yes	1 (6.7)	0	
No	14 (93.3)	7 (100)	

All numerical values as Mean ± S.D. Chi-squared test was used for categorical variables

*P-values are significant (< 0.05)

## Discussion

We aimed to assess the survival outcomes in cirrhotic patients presenting with concomitant ACS and AVB and the role of anti-platelet agents. Our study has demonstrated high short-term mortality (24.1%) amongst patients with concomitant AVB and ACS, with increased severity of underlying liver disease in fatal patients. Moreover, the use of anti-platelet regimes was not significantly associated with either follow-up variceal bleeding or cardiac events.

Our mortality rate is in concordance with published literature, which provides evidence of increased risk, as well as higher mortality rates of ACS in patients with underlying cirrhosis [18–20]. Wu VC et al. found a significantly higher all-cause mortality in AMI patients with cirrhosis, compared to those without cirrhosis (hazard ratio [HR] 1.30, *P* < 0.001), with higher incidences of complications such as variceal bleeding, hepatic encephalopathy, and ascites (HR 2.27, *P* < 0.01) [21]. Recently, the impact of cirrhosis in PCI was explored, showing more than a 2-fold increase in Index mortality (OR 2.22, *P* < 0.01) and gastrointestinal bleeding (OR 2.52, *P* < 0.01), although variceal bleeding did not have any significant association with Index mortality (OR 2.04, *P* = 0.14) [22].

Albumin, significantly lower in our deceased patient population (*P* = 0.045), has been described as a significant predictor of death in cirrhotic patients [23, 24]. In addition, although ascites also did not have a significant impact on mortality (*P* = 0.215) in our patients, it was the most common sign of decompensation (526.6%). Ascites is usually the landmark sign in progression into the decompensated phase of cirrhosis, and depicts a worsening prognosis of CLD, with mortality estimated to be 50% in 2 years [25–27].

UGB was the predominant complaint in most of our patients (86.0%). It is most frequently caused by AVB amongst cirrhotic patients and has been strongly associated with mortality in the presence of ACS [13, 28–30]. Our patients were managed with single or multiple sessions of variceal band ligation, which is the optimal initial management for bleeding esophageal varices due to decreased re-bleeding and higher safety profiles [31, 32]. Of not, our overall hospital’s internal audit data have demonstrated the short-term (1 month) recurrent AVB rate to be < 1% after successful band ligation with no concomitant ACS or antiplatelet use. Only 1 deceased patient in our study required the use of TIPS, which has been shown to significantly reduce treatment failure (*P* = 0.001) and mortality (*P* = 0.01) [33]; however, precaution is advised due to cardiovascular complications amongst cirrhotics, likely attributable to diastolic dysfunction [34].

Our results have demonstrated that the use of SAPT is not significantly associated with higher rates of recurrent variceal bleeding (*P* = 1.000), and neither with recurrent cardiac events (*P* = 0.091), at 6 months of follow-up. Interestingly, 2 patients from the group treated with SAPT developed a recurrence of ACS, as compared to no recurrence of cardiac events in patients not receiving antiplatelet therapy. We postulate this to be attributable to various confounding factors that may have been present in those individual patients such as male gender, age, severity, site, and extent of luminal narrowing, and varied compliance to medications. Furthermore, SAPT has been shown to demonstrate a significantly higher incidence of ischemic events, and cardiovascular mortality as compared to DAPT [35]. However, conclusive inferences cannot be made with our limited 6-month follow-up, and we urge the need for prospective studies with a longer observation period on a larger sample population to provide better insight into the impact of anti-platelet agents amongst these high-risk patients.

Moreover, we could not assess the use and comparison of DAPT. A recent study reported that cirrhotics with acute MI on DAPT had significantly increased UGB compared to non-cirrhotics (28.0 vs. 20.2%: SHR 1.49, 95% CI = 1.31–1.70). This study also compared outcomes between SAPT and DAPT. Similar risks of gastrointestinal bleeding were identified from both DAPT and SAPT (37.8% vs. 28.0%; SHR 1.05, 95% CI = 0.82–1.35), with a significantly decreased all-cause mortality (32.7% vs. 58.5%; HR 0.77, 95% CI = 0.63–0.94). There were no significant differences in all-cause mortality, recurrent MI, or bleeding, depending on the use of either Clopidogrel or Aspirin as SAPT [36]. Additionally, Roula et al. describe the role of anticoagulation in cirrhosis for variceal bleeding. Overall increased risk of bleeding within 6 months of initiation of anticoagulation was associated with the use of antiplatelets and warfarin (both *P* < 0.05). However, the only predictor of bleeding in cirrhotic patients on anticoagulation was a history of esophageal varices (OR 5.7, *P* < 0.05) and not the use of antiplatelet therapy (OR 1.8, *P* = 0.121), theorizing that increased bleeding in cirrhotic patients is due to pre-existing esophageal varices [37]. Thus, we suggest anti-coagulation as SAPT is safe since it does not seem to increase the risk of variceal bleeding on short-term follow-up; however, the physicians’ discretion, patient comorbidities, and severity of the liver disease must be considered and accounted for, during initiation of this therapy.

Our study has several limitations. Since this was only a single-center study, with small sample size, external validity may be compromised. Additionally, inherent limitations of the retrospective nature of the study are present, such as the accuracy of medical documentation and missing information. We were also unable to perform a regression analysis to demonstrate the risk factors responsible for the death of patients at index admission. Perhaps the most significant limitation of our study was that cardiac catheterization was not performed to assess the presence of occlusive CAD. Deferring PCI may have been attributed to the critical condition of patients at the time of Index admission, with the risk of potentiating variceal bleeding.

## Conclusion

In summary, we conclude that concomitant AVB and ACS is a severe disorder amongst cirrhotic patients, with a high mortality rate. Provided the patient survives the initial insult, the use of single anti-platelet agents does not seem to exacerbate the incidence of variceal bleeding on follow-up, given successful obliteration of varices is achieved using band ligation. Furthermore, we hope our study serves as the steppingstone for large prospective clinical trials and multi-centric studies, to help delineate the efficacy and risks of using antiplatelet agents for this life-threatening presentation of events.

## Data Availability

The datasets used and/or analyzed during the study are available upon reasonable request.

## Abbreviations

ACS: Acute coronary syndrome
AKUH: Aga Khan university hospital
AMI: Acute myocardial infarction
ASA: Acetyl-salicylic acid
ACLF: Acute on chronic liver failure
CAD: Coronary artery disease
DAPT: Dual anti-platelet therapy
EVBL: Esophageal variceal band ligation
NSTEMI: Non-STEMI
PCI: Percutaneous coronary intervention
SAPT: Single antiplatelet agent
STEMI: ST-Segment elevation myocardial infarction
TIPS: Trans-jugular intrahepatic porto-systemic shunt
UGB: Upper-gastrointestinal-bleeding
